# Humanely Ending the Life of Animals: Research Priorities to Identify Alternatives to Carbon Dioxide

**DOI:** 10.3390/ani9110911

**Published:** 2019-11-02

**Authors:** Aline R. Steiner, Shannon Axiak Flammer, Ngaio J. Beausoleil, Charlotte Berg, Regula Bettschart-Wolfensberger, Rebeca García Pinillos, Huw D.R. Golledge, Michael Marahrens, Robert Meyer, Tobias Schnitzer, Michael J. Toscano, Patricia V. Turner, Daniel M. Weary, Thomas C. Gent

**Affiliations:** 1 Department of Clinical and Diagnostic Services, Section of Anaesthesiology, Vetsuisse Faculty, University of Zurich, Winterthurerstrasse 258c, 8057 Zurich, Switzerland; asteiner@vetclinics.uzh.ch (A.R.S.); rbettschart@vetclinics.uzh.ch (R.B.-W.); 2 Department of Clinical Veterinary Medicine, Section of Anesthesia and Analgesia, Vetsuisse Faculty, University of Berne, Laenggassstrasse 124, 3012 Bern, Switzerland; shannon.axiak@vetsuisse.unibe.ch; 3 Animal Welfare Science and Bioethics Centre, School of Veterinary Science, Massey University, Palmerston North 4410, New Zealand; N.J.Beausoleil@massey.ac.nz; 4 Department of Animal Environment and Health, Swedish University of Agricultural Sciences, Box 234, SE-53223 Skara, Sweden; Lotta.Berg@slu.se; 5 Animal and Plant Health Agency and Department for Environment, Food and Rural Affairs, Nobel House, 17 Smith Square, London SW1P 3JR, UK; 6 Universities Federation for Animal Welfare (UFAW), The Old School, Brewhouse Hill, Wheathampstead, Hertfordshire AL4 8AN, UK; golledge@ufaw.org.uk; 7 Institute of Animal Welfare and Animal Husbandry, Friedrich-Loeffler-Institut, Dörnbergstraße 25/27, 29223 Celle, Germany; Michael.marahrens@fli.de; 8 Department of Clinical Sciences, College of Veterinary Medicine, Mississippi State University, Mississippi State, MS 39762, USA; robert.meyer@msstate.edu; 9 Roche Pharma Research and Early Development, Pharmaceutical Sciences, Roche Innovation Center Basel, F. Hoffmann-La Roche Ltd., Grenzacherstrasse 124, 4070 Basel, Switzerland; tobias.schnitzer@roche.com; 10 Center for Proper Housing: Poultry and Rabbits (ZTHZ), Animal Welfare Division, VPH Institute, University of Bern, 3052 Zollikofen, Switzerland; michael.toscano@vetsuisse.unibe.ch; 11 Department of Pathobiology, University of Guelph, Guelph, ON N1G 2W1, Canada and Charles River, Wilmington, MA 01887, USA; Patricia.Turner@crl.com; 12 Animal Welfare Program, University of British Colombia, 2357 Main Mall, Vancouver, BC V6T 1Z4, Canada; dan.weary@ubc.ca

**Keywords:** animal welfare, carbon dioxide, stunning, killing, euthanasia, rodents, poultry, pigs, aversion, air hunger

## Abstract

**Simple Summary:**

Carbon dioxide is commonly used for stunning animals prior to killing. It allows several animals to be killed at once, reduces the need for handling, and is a reliable method. However, research in laboratory rodents, poultry, and pigs has indicated that it causes considerable aversion at concentrations above ambient conditions. Currently, there are no available alternatives with desirable characteristics. This manuscript describes a list of research priorities to find and implement the use of alternative methods or agents to improve animal welfare.

**Abstract:**

The use of carbon dioxide (CO_2_) for stunning and killing animals is considered to compromise welfare due to air hunger, anxiety, fear, and pain. Despite decades of research, no alternatives have so far been found that provide a safe and reliable way to induce unconsciousness in groups of animals, and also cause less distress than CO_2_. Here, we revisit the current and historical literature to identify key research questions that may lead to the identification and implementation of more humane alternatives to induce unconsciousness in mice, rats, poultry, and pigs. In addition to the evaluation of novel methods and agents, we identify the need to standardise the terminology and behavioural assays within the field. We further reason that more accurate measurements of consciousness state are needed and serve as a central component in the assessment of suffering. Therefore, we propose a roadmap toward improving animal welfare during end-of-life procedures.

## 1. Definitions

**Abnormal respiratory pattern**—any deviation from what is considered the normal physiologic range in that species in that environment (e.g., tachypnoea, apnoea, irregular rhythm, shallower or deeper inhalation, dyspnoea). Refers to the physical respiratory activity that can be observed and not to associated experiences/sensations [1,2].

**Air hunger**—the result of the discrepancy between the drive for respiratory and lung inflation [3]. Reported as the most unpleasant component of dyspnoea by humans, and even at moderate levels is more unpleasant than maximal respiratory effort [4].

**Anxiety**—a negative emotional state characterised by anticipation of real or potential threats, typically enhances environmental scanning [5].

**Apnoea**—absence of breathing [6].

**Aversion**—“avoidance of a stimulus, situation or behaviour” [7]. Refers to the theoretically beneficial behaviours expressed to avoid or withdraw from potentially noxious stimuli or situations rather than to the underlying mental or affective status. Aversion, per se, does not indicate that an animal is suffering; however, suffering may result from inescapable exposure to aversive stimuli or situations [7,8].

**CAS**—controlled atmosphere stunning. Any method changing inspired gas concentrations or ambient pressure in a way that leads to unconsciousness [9,10].

**Chest tightness**—a sensation described by humans upon bronchoconstriction [3].

**Distress**—unlike stress, distress is “an aversive, negative state in which coping and adaptation processes fail to return an organism to physiological and/or psychological homeostasis” [11].

**Dyspnoea**—“laboured” breathing with observable respiratory effort [6] (intended use in this document). In human medicine, the term dyspnoea describes the unpleasant sensation of air hunger and/or increased respiratory effort and/or chest tightness [3]. In humans, laboured breathing can occur without the sensation of dyspnoea and vice versa [3].

**Euthanasia**—literally “good death”; defined as “ending the life of an individual animal in a way that minimises or eliminates pain and/or distress” [12]. Controversy exists whether use of the term “euthanasia” additionally requires that animals are killed in accord with their own interests and/or because killing would relieve an unacceptable welfare state (e.g., [6] versus [12]). For this document, euthanasia is defined as killing that minimises welfare impacts and is in accord with an animal’s own interest; for example, the killing of sick animals or when a humane endpoint is reached in an experiment. Euthanasia is not used to describe death solely for the purposes of meat consumption (slaughter), the depopulation of surplus healthy animals, or to harvest tissues from healthy animals for data collection.

**Fear**—a response to a present threat, typically initiates behaviours to negate the situation [5,13].

**Gasping**—describes two different situations: Either, a breathing pattern caused by extreme hypoxia [partial pressure of arterial oxygen (PaO_2_) <5 mmHg], that is potentially life-saving (restores PaO_2_ to 30 mmHg [14], produces cardiac output during cardiac arrest [15], and increases survival in humans with cardiac arrest [16]); otherwise, it is called agonal, and occurs under circumstances in which consciousness has typically already been lost [14,16]. Alternatively, it describes deep inhalations with or without open mouth/bill and extended neck that is observed in conscious animals exposed to carbon dioxide (CO_2_) (i.e., before loss of posture) [17,18].

**Killing**—general term for inducing death, regardless of the purpose and the method used [12,19].

**LAPS**—low atmospheric pressure stunning. A CAS method gradually reducing ambient pressure and thereby partial pressure of oxygen, i.e., causing hypobaric hypoxia [9].

**Nociception**—the detection, transmission, and processing of noxious (i.e., harmful) stimuli by nociceptors, to and within the central nervous system. Nociception refers to the physiologic basis required but not sufficient for perception of pain; however, it can occur in the absence of consciousness where pain is absent [6].

**Pain**—“an unpleasant sensory and emotional experience associated with actual or potential tissue damage, or described in terms of such damage” [20].

**Respiratory effort**—an experience relating to the amount of respiratory muscle force that is currently applied to generate ventilation. In humans, conscious awareness of respiratory effort arises when the contribution of voluntary motor command to overall respiratory muscle drive increases [2]. The sensation becomes unpleasant when the motor command needed to elicit a given level of ventilation is greater than normal, which may occur in animals with respiratory disease.

**Stress**—“real or perceived perturbation to physiologic homeostasis or psychologic well-being”, effects can be “positive, negative, or inconsequential”; i.e., stress does not necessarily involve a negative emotional component [11,21].

**Stunning**—inducing unconsciousness prior to killing. The European Council uses the terms “simple stunning” when a loss of consciousness is reversible, and “stunning” when a loss of consciousness is irreversible [22]. However, in this document, this differentiation will not be made.

**Superficial/deep breathing**—smaller/larger tidal volume than at resting baseline conditions.

**Tachypnoea**—increased respiratory rate [6].

**Tonic Immobility Test**—a test to determine fear levels in poultry. Birds are repeatedly restrained manually for a short period (typically 15 s) to simulate predation until they remain immobile after release of restraint for at least 10 s, i.e., “feign death”. Number of attempts required to induce immobility and total time of immobility are assessed; fewer attempts needed and a longer duration of immobility are interpreted as higher fear levels [23].

**Welfare (state)**—the state of welfare of an animal can be positive, negative, or neutral, depending on the total of positive and negative affective states experienced within the relevant time period [24].

## 2. Introduction

**Figure 1:**
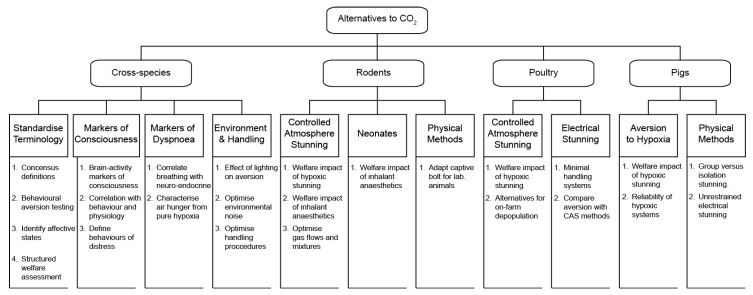
Structural overview of research priorities.

This research strategy is an initiative of the Swiss Federal Food Safety and Veterinary Office (FSVO) aimed at determining research priorities to identify and implement alternative stunning methods for live animals, specifically: mice, rats, pigs, and poultry. Whilst specific issues relating to carbon dioxide are discussed, it is not intended to be a comprehensive literature review on carbon dioxide stunning. Furthermore, this article is not an exhaustive list of research questions. Here, we focus on identifying humane methods to render animals unconscious prior to killing, rather than the methods of killing (Figure 1).

An ideal stunning or euthanasia method should first reliably induce loss of consciousness without causing distress and persist until death occurs (due either to the stunning method or to a secondary killing method applied after loss of consciousness). Furthermore, the chosen stunning method should be compatible with the purpose of the animal, (e.g., residual-free when animals are killed for meat consumption) and also compatible with subsequent investigations when laboratory animals are killed [12]. Of secondary importance, an ideal method should be both physically safe, easy to use and have low psychological impact for the human operator [25]. Furthermore, it should be cost-effective and not environmentally damaging [12,26].

Carbon dioxide (CO_2_) is one of the most commonly used agents for stunning prior to killing rodents [7,21], pigs [27], and poultry [28]. Controlled atmosphere (or non-physical) stunning (CAS) methods are attractive since they require a minimal physical restraint of animals, thereby reducing direct interaction between animals and the operator, which may be a cause of stress. CO_2_ is cheap, and systems can be used without extensive training [21]. Furthermore, it is denser than ambient air, making it easier to contain in unsealed vessels. It reliably produces unconsciousness and death in a concentration-dependent manner. However, its use is controversial due to reports of impacts on animal welfare, including aversion, which may reflect negative affective states such as fear, pain, and air hunger [7].

CO_2_ exposure induces generalised intracellular acidosis, which depresses basal and evoked neural activity, and thereby induces unconsciousness [29]. Depending on CO_2_ concentrations, species, and the method of assessment, loss of consciousness occurs 26 to 150 s after an onset of exposure (rats: 30 to 150 s [30,31]; mice: 51 to 75 s [32]; pigs: 33 to 60 s [33,34]; poultry: 20 to 26 s [9,35]). CO_2_ exposure has been shown to be aversive in rats [36,37,38,39], mice [32,40,41], pigs [42,43], and poultry [44,45,46], to cause abnormal breathing patterns commonly interpreted as air hunger (rats [18], mice [32], pigs [33], poultry [1], humans [47]) and to elicit anxiety [18,48], which is in line with reports of increased anxiety or even panic attacks in humans [49,50,51]. High concentrations of CO_2_ elicit pain in humans [52] and activate nociception pathways in rats [53,54] and poultry [55]. Therefore, animals are highly likely to experience pain and distress prior to loss of consciousness.

## 3. General Aspects (All Species)

Minimising the welfare impacts of rendering an animal unconscious prior to killing requires a great deal of species-specific consideration [7]. In this manuscript, we detail these considerations in sections devoted to each of the species groups, namely rodents, poultry, and pigs. However, we also identified that some considerations prevail across all species. In this first section, we will discuss these multi-species issues before moving on to discuss species specifics.

### 3.1. Standardised Terminology, Investigation of the Animal’s Experience, and Structured Welfare Assessment

#### 3.1.1. Statement of the Problem

A comparison of findings between studies in the same species is frequently hampered by variability in descriptions of behaviours and an inconsistent use of terms to describe them. This variability is problematic, as the description and measurement of indicator variables influences how they are interpreted as reflecting impacts on welfare. For example, CO_2_ commonly induces inspiration of an increased tidal volume in poultry, while the bill may be open, and the neck extended. This pattern is variably described as “gasping” [17,56,57,58], “deep breathing” [59], or “respiratory disruption” [1,44], which has variably been interpreted as distress.

Definitions and the usage of more complex terms such as aversion, anxiety, and distress vary, as do techniques to measure them. For example, aversion has been assessed by place preference testing [39], escape behaviours [40], vocalisations [43,60], and physiological parameters during forced exposures [61]. Terms and definitions may be used interchangeably and sometimes incorrectly; for example, stress and distress are often used to describe each other despite having different meanings (see definitions and ref: [62]). Aversion behaviours are often interpreted as signs of distress; however, they may not necessarily indicate distress if the animal can successfully escape the aversive agent [7].

There are significant challenges associated with the use of both physiological and behavioural methods to infer the unpleasant affective experience of pain. Pain, or the absence of, is sometimes inferred from physiological parameters that are non-specific [63]. Although it cannot be proven that animals experience the negative emotional states associated with pain, it is generally assumed that procedures that are painful for humans are also painful for animals [8]. Based on this assumption, behavioural indicators of pain have been identified, and species-specific pain scales validated [64,65,66]. A caveat with pain scales is that facial expressions and behaviours relying on motor function may be impaired during stunning, and sensitivity may also be agent-specific, meaning that each pain scale must be validated for each context, making comparisons between methods difficult [64]. Furthermore, most pain scales have been developed for assessing post-surgical pain, and are therefore unlikely to be useful in association with stunning [67]. They are also notoriously inaccurate for very short-term pain expression. Other monitoring indicators of pain and distress commonly used in abattoirs include vocalisation and falling [68].

The comparison of different stunning methods across studies requires a systematic and fully documented approach to assess the overall pain, suffering, and distress caused, as well as practical aspects such as repeatability and operator-related aspects.

#### 3.1.2. Research Priorities

1 Standardise the use of terminology by means of a consensus list of definitions and clarify underlying mechanisms, where required, for unambiguous definitions. Especially for poultry, a thorough characterisation of respiratory patterns and behaviours is required to allow cross-study comparison, as those are described, categorised, and interpreted differently across studies [9]. However, inconsistent use of terminology is an issue in all species covered by this strategy.

2 Standardise behavioural and physiological testing for aversion. Aversion has been investigated by observing spontaneous or conditioned behavioural, physiological reactions, or biochemical parameter reactions to a stimulus. For both approaches, formulating hypotheses about the expected response or behaviour increases the strength of evidence that can be obtained [8]. Whilst a battery of behavioural tests for aversion are validated in rodents (e.g., [69,70]), variable approaches have been used to assess aversion in poultry [17,44,46] and pigs [27,42,43]. Therefore, robust aversion tests should be developed and validated in these species.

The overall experience of an animal may be tested using a reward that has to be relinquished in order to escape an aversive condition, or by offering an aversive environment (e.g., a brightly lit space in rodents) as an alternative into which to escape. Alternatively, the stimulus in question can be paired with a certain environment and conditioned place preference/aversion (compared to an alternative stimulus) measured in the absence of any stimuli [8].

3 Formulate robust methods to identify specific negative affective experiences such as pain, fear, and anxiety, and to grade their intensity.

Assessing the welfare impact of an intervention or condition ultimately depends on identifying affective states an animal experiences in the relevant period of time (e.g., [24,71]). In the absence of clear evidence for the type of affective experience, observations are often interpreted on untested assumptions (e.g., perception of an inactive animal as less stressed than an overactive one). While the analogy argument is useful to posit the presence of felt emotions in animals, more sophisticated methods are required to identify the type and intensity of affective experience in a specific situation.

To address contributions of specific affective states to the overall experience, several approaches can be used. The first would be to gain a comprehensive understanding of the effects of the stimulus or condition on the physiology and neurophysiology of the animal. This, evaluated in conjunction with an understanding of the human physiology and neurophysiology and reported affective experience, informs what affective states (and thus observed responses) should be investigated in the behavioural tests. Subsequently, behavioural tests developed to assess a specific component, e.g., anxiety, can be applied during and/or immediately after exposure to the stimulus in question, such as the open field test [23,72], light/dark test, and social interaction test in rodents [73,74], or tonic immobility in poultry [23]. For rats, it was shown that thigmotaxis in the open field test was increased and social interaction reduced immediately after exposure to 20% CO_2_ [18], indicating increased anxiety, but similar testing was not performed for putative alternative methods. No known data exists in poultry or pigs investigating fear or anxiety induced by any of the stunning methods. Overall, the systematic investigation of anxiety caused by (elements of) different stunning methods is lacking. Additionally, pain scoring systems suitable for the assessment of acute pain should be developed and validated for all species. For example, it is challenging to assess whether low atmospheric pressure stunning (LAPS) causes colic pain in broiler chickens [9], when no validated pain scoring is available.

A complementary approach to investigate the contribution of specific affective states during stunning is to examine the modulating effects of drugs that are known to reduce anxiety, dyspnoea, or pain, on behavioural and physiological responses [8]. Likewise, using drugs known to induce a specific negative affective state, e.g., anxiogenics, animals can be trained to perform specific behaviours in that state, expecting that they will generalise this behaviour when the same negative affective state is caused by a different stimulus [8].

An inherent challenge is that different stunning methods elicit different negative affective experiences due to their different physiological effects. Therefore, behavioural indicators to assess certain negative affective experiences cannot be used universally. For instance, behavioural tests used during controlled atmosphere stunning (CAS) are not applicable to physical methods due to differences in exposure times.

Correlating behavioural responses with physiological measurements of endocrine and neurophysiological parameters will validate the interpretation of behavioural responses and facilitate comparisons of different stunning methods. However, physiological parameters should only be measured and interpreted in conjunction with behavioural evaluations of aversion because some methods (e.g., CAS with CO_2_) can elicit physiological changes that may not reliably reflect conscious experiences (e.g., measurably raised plasma cortisol levels or respiratory lesions may occur only after loss of consciousness) [21].

Integrating the different types of negative affective experiences caused by a specific stunning method into an overall assessment and comparing the welfare impact of different methods can nevertheless be challenging, and the following paragraph focuses on approaches to this challenge.

4 Develop a structured, systematic welfare assessment specifically for stunning, taking into account all potential sources of distress, including those occurring in a minority of animals, their severity, and duration. Distress is an umbrella term for strongly negative affective experiences that arise when stress is continual or cannot be compensated for. In the context of stunning, different types of stressors are involved and elicit different types and intensities of negative affective experiences (e.g., pain, air hunger) depending on the method or agent [9]. For a thorough comparison of methods, the type and intensity of negative affective experiences should be identified, as should the temporal progression of the intensity, as well as the duration prior to the loss of consciousness on both an individual and population level [75]. A descriptive ranking to assess killing methods has previously been established [76]. Importantly, this system does not employ numerical scaling, and therefore negates quantitative comparisons of severity. Detailing exactly how information about the quality, intensity, duration, and progression should be weighed to give an overall assessment is beyond the scope of this document, as is whether such a tool should directly compare one method with another, or simply classify it as “acceptable” versus “unacceptable”. Importantly, the terms ‘structured’ and ‘systematic’ should not be understood as quantitative in this context, although quantifiable comparisons may be included in an assessment.

### 3.2. Define Markers of (Un)Consciousness

#### 3.2.1. Statement of the Problem

For an animal to experience distress, it must be conscious [7]; therefore, an accurate determination of consciousness is required to assess welfare impacts. The exact point at which awareness to external stimuli is abolished is unknown [77,78]. Current behavioural surrogates for loss of consciousness (e.g., loss of righting reflex, loss of posture, loss of motion, loss of response to a stimulus) are variably correlated with a supposed loss of consciousness [79]. They rely on a degree of responsiveness requiring a motor output that may be impaired or rendered absent by the procedure [80,81]. Unresponsiveness to external stimuli (e.g., loss of righting reflex) does not preclude awareness, and furthermore, responses based on spinal or cranial nerve reflexes, (e.g., pedal withdrawal or corneal reflex) can occur in the absence of consciousness [82]. Therefore, such reflexes may not be reliable indicators of a consciousness state in the context of stunning. In the context of CAS, brain-wide electroencephalography (EEG) is used to infer loss of consciousness, often by the onset of slow-frequency high-amplitude waves, although these may also occur during wakefulness, as demonstrated in rodents [83] and humans [84]. Furthermore, the EEG does not reveal the absence or presence of “information transfer” within the brain that is required for awareness to stimuli [85]. Depth of anaesthesia monitors such as the bispectral index (BIS) aggregate EEG and muscle activity parameters into a single score. Whilst these monitors may reliably indicate internal brain state in humans once anesthetised, they are notoriously inadequate for determining loss of consciousness [86]. A complete absence of sensory evoked potentials (EPs) is definitive for the lack of awareness to external stimuli; however, they are highly modality-specific (e.g., auditory versus visual versus somatosensory), and EPs may be present in animals that are deeply anesthetised [87]. In humans, EPs are correlated with awareness to a stimulus [88] or in response to verbal commands [89]; however, this cannot easily be performed in animals. Furthermore, anaesthetic agents diminish EPs in a continuous rather than binary manner, and currently there is no definition as to where in that continuum sensory awareness is lost. This is compounded by the observation that certain non-executive sensory brain circuits remain electrically responsive to sensory stimulation during deep anaesthesia [90]. Further to this, EPs are not informative about ongoing internal sensations such as pain and allodynia.

The accurate assessment of state of consciousness is important both for the reporting of studies aimed at refining stunning methods, as well as for the practical assessment of the welfare impact in the laboratory or abattoir environments.

#### 3.2.2. Research Priorities

1 Identify brain activity-based markers for loss and regain of consciousness. The European Food Safety Authority panel’s “Guidance on the assessment criteria for applications for new or modified stunning methods regarding animal protection at the time of killing” [10] states that the “normal functioning of neurons in the thalamus and cerebral cortex or analogous structures” is a necessary condition for consciousness and considers EEG as the gold standard to demonstrate a disruption of normal function. However, a more detailed characterisation of this “normal function” or of suitable EEG measures is not provided. Given that in human clinical anaesthesia, EEG-based monitoring is currently unable to detect the presence or absence of awareness [91], further search for brain activity-based markers of consciousness is warranted, and the inclusion of additional methods to assess brain activity, e.g., functional MRI, may be required.

2 Correlate these markers with behavioural and gross physiological endpoints. The assessment of loss of consciousness during a general use of stunning methods is important to the development and implementation of studies on stunning methods. Such validation methods as electrophysiology (e.g., EEG, EMG, ECG, EPs) are unlikely to be of practical use in the laboratory or abattoir setting; therefore, reliable behavioural surrogates are required to allow successful monitoring.

3 Determine which behavioural endpoints can be reliably used to report investigation findings and assess animal welfare in a practical setting. The measurement of brain activity requires special equipment and skills and may in some cases not be compatible with the stunning method under investigation. Establishing clinically accessible markers/proxies for brain activity-based markers of (un)consciousness would help to standardise the reporting of investigations of stunning methods, facilitate the assessment of distress caused by specific methods, and allow a more precise assessment of animal welfare in practical settings.

### 3.3. Validate Markers of Dyspnoea, Particularly Air Hunger, during Hypercapnic and Hypoxic Stunning

#### 3.3.1. Statement of the Problem

Humans exposed to hypercapnia and/or intense hypoxia often report a sensation of air hunger [3,92]. In the context of CAS, air hunger might arise when an elevated drive to breathe cannot possibly be satisfied by increased respiration, even in healthy animals, i.e., the harder/faster the animal breathes, the more hypercapnic/hypoxic it becomes, and the greater the drive to breathe. In animals with pre-existing conditions that impair their ability to mount a respiratory response, the intensity of any air hunger occurring during hypercapnic/hypoxic stunning will be exacerbated, i.e., lung inflation will be further mismatched to drive. The experienced air hunger is consequently not necessarily linearly correlated to observed respiratory rates and depth.

Abnormal respiratory patterns are described in rodents [21], pigs [27,43], and poultry [1] exposed to hypercapnic, hypoxic, as well as combined hypercapnic/hypoxic conditions. It remains uncertain at which point these behaviours change from indicators of adaptive compensation (i.e., increased respiration to satisfy enhanced drive) to indicators of distress (i.e., air hunger due to unsatisfied respiratory drive). Importantly, unpleasant respiratory sensations other than air hunger might also occur during exposure to higher than normal CO_2_ atmospheres; for example, humans report feelings of choking and suffocation after a single breath, which cannot be ascribed to enhanced arterial CO_2_ levels [93].

Post-mortem examinations additionally demonstrate pulmonary lesions such as haemorrhage and oedema in rats [94] and mice [61,63] stunned with CO_2_ and inert gas methods. If these occur before loss of consciousness, such lesions could impair respiratory function, thereby causing or exacerbating air hunger. Therefore, abnormal respiratory patterns and lung lesions are insufficient to assess the nature and degree of distress experienced by the animals.

#### 3.3.2. Research Priorities

1 Correlate respiratory variables to neurophysiologic and endocrine measurements, aversion testing, and utilise pharmacologic interventions to elucidate relations between observable respiratory patterns and affective states. Respiratory patterns should be characterised during the course of exposure to identify changes occurring prior to loss of consciousness. It should also be determined at which timepoint lung lesions develop. Combining the measurement of the physiological stress response with aversion testing will help elucidate the effects of different gas concentrations and pressures, and aid the integration of findings from previous studies measuring the physiological stress response to similar interventions. Pharmacologic interventions with methods known to relieve air hunger in humans, such as inhaled furosemide [95] or anxiolytics [3], may elucidate the components of dyspnoea that cause distress.

2 Investigate air hunger caused by pure hypoxia and strategies to minimise the duration and degree of associated distress. Hypoxia-inducing CAS methods are prominently proposed alternatives to CO_2_ [41,96]. Although ultimately air hunger comes from a mismatch between the drive for respiratory motor activity and lung inflation, hypercapnic and hypoxic responses involve different chemoreceptors in ascending pathways [2]. The correlation between respiratory patterns and associated experiences may consequently be specific for each condition. For example, gasping is commonly reported with both CO_2_ and inert gas stunning in mice and rats [21], poultry [45,56], and pigs [33,97]; however, whilst gasping due to hypercapnia commonly occurs prior to a loss of posture, gasping due to inert gas hypoxia is often considered agonal (see definitions). Therefore, the extent of air hunger caused specifically by hypoxic CAS methods should be characterised, and flow as well as decompression rates should be optimised to minimise the distress caused by air hunger.

### 3.4. Refinement of Handling and Environment

#### 3.4.1. Statement of the Problem

Handling and environmental factors prior to and during stunning add to the overall experience and affect how much stress animals experience during the stunning process. Pigs and poultry arriving at the abattoir are typically food deprived, removed from their familiar environment, and transported, and may therefore already experience a degree of short-term stress [98,99]. In rodents, pre-existing cumulative stress from procedures or experimentation may be more variable (e.g., animals at the end of an experiment versus surplus animals). Despite species-specific differences, most animals experience stress resulting from light, noise, the grouping of animals, and handling by humans.

#### 3.4.2. Research Priorities

1 Determine the influence of lighting conditions on stress and aversion in general and on time to unconsciousness for all CAS methods. Rats and mice find bright light aversive [73,100,101], and mice show significantly less anxious behaviours in a darkened chamber compared to a brightly lit chamber during CO_2_ killing [102]. In poultry, illumination promotes active behaviours, whereas darkness promotes sleep [103]. Accordingly, when LAPS is applied in the dark, time to loss of consciousness is shorter, and time to deep inhalation, a suggested indicator for air hunger, is longer in poultry broilers [103]. Pigs tend to move from darker to brighter areas [104], which may facilitate handling prior to stunning; however, how lighting affects the stunning is unknown.

Investigations of variable lighting conditions, including intensity and wavelength, are needed to determine stress reduction regardless of the stunning method. Additionally, investigations of whether low light may reduce the time to unconsciousness for CAS methods are needed. Optimal lighting conditions, which minimise stress whilst providing safe and efficient working conditions, should be identified for each species.

2 Develop equipment and optimise processes to minimise environmental noise as well as noise caused by stunning equipment. Noise causes a stress response in rodents [101]; additionally, poultry exhibit increased plasma cortisone levels [105], and pigs show increased grouping behaviours [99] when exposed to pre-recorded abattoir noise. Therefore, strategies should be developed/adopted to reduce noise in the lairage and stunning areas of abattoirs as well as noise associated with the stunning method per se. A special consideration with rodents is that rats and mice are able to hear frequencies in the ultrasonic range [106]. Therefore, equipment should be designed to operate without producing ultrasonic noise. Further potential refinements include auditory environmental enrichment, which is considered to reduce stress in multiple species [107].

3 Optimise grouping and handling procedures prior to and during killing. Rodents, poultry, and pigs are social species, and the presence of conspecifics ameliorates stress and anxiety in social animals [108]. These species are usually unacclimatised to regular handling, and therefore, these practices should generally be considered as a stressor.

Stunning practices are most variable in rodents, and largely depend on the situation in which they are used, including experimental requirements, local regulations and practices, and population size [7]. Mice killed with CO_2_ in groups had lower plasma catecholamine concentrations than mice killed individually [109]. Single rats killed with CO_2_, showed no significant difference in physiological variables, and there were only minor behavioural differences between animals killed in their home cage and animals killed in an induction chamber [110]. The stress-alleviating effect of killing animals in groups remains to be confirmed for rats and for other CAS methods. Whether killing in the home cage generally does not provide a measurable stress reduction or whether the stress of CO_2_ killing masked beneficial effects of home cage killing also remain to be determined.

Poultry are typically stunned in groups when CO_2_ is used or remain in sight of other birds when electrical stunning is used. In the presence of other birds, animals are more alert during exposure to CO_2_, argon (Ar), or a combination of these [1]. Nevertheless, several behaviours indicative of stress have longer latencies to first occurrence or shorter durations and/or less bouts in birds undergoing LAPS in groups, indicating a beneficial effect of group stunning [1,111]. Only latencies for slow wing flapping (i.e., prior to loss of posture) were shorter in groups, which was possibly because birds were disturbed by wing-flapping neighbours [111]. Optimal stocking densities for CAS methods should be determined and electrical stunning processes adapted to minimise the stress associated with handling.

Little data exists in pigs to demonstrate the benefits of single versus group stunning. Electrical stunning is sometimes performed in a race that animals enter one at a time; however, the use of electrical prods adds to the aversion of this method [112]. Social isolation in pigs is considered stressful, and the optimisation of group sizes for stunning for CAS methods and the feasibility of electrical stunning in group pens should be investigated.

## 4. Rodents

### 4.1. Rodents-Introduction

Killing by gradual fill (10%–30% chamber volume per minute) CO_2_ exposure is the currently recommended practice [12]. This method is commonly used at the end of experimentation, for sick and injured animals, for surplus animals, and for feed animals in zoos [7]. Recommended and practically used chamber replacement rates vary widely [21]. It is thought that slow displacement rates induce unconsciousness at CO_2_ concentrations below the threshold for pain [113], although the absence of pain in this regard has not been proven.

Mice and rats are sensitive to detecting low O_2_ or high CO_2_ concentrations [37,114,115,116]. Both mice [115] and rats [36,37,39,117] will chose to escape from chambers at 12%–18% CO_2_ either despite the presence of a food reward or if the alternative is a brightly lit open space, which they would normally avoid, thus indicating the degree of aversion [118,119]. Animals unable to escape will show a range of avoidance behaviours including rearing, pushing the lid of the chamber, jumping, freezing, and face grooming, and show physiological indicators of stress [18,40,41,61,109,117,120,121]. Rats show gasping at around 10% CO_2_ [18], and laboured breathing occurs in mice after only 14 s of CO_2_ exposure at flow rates as low as 20% chamber replacement per minute [32]. Whether gasping is merely a physiological response to CO_2_ or associated with an experience of air hunger as reported by humans at similar concentrations of CO_2_ (8% and above) is unknown. A freezing response, which is accepted as the manifestation of fear in rodents, is elicited upon exposure to 10% CO_2_ [18,48], which is mediated (among others; see [122]) by a number of mechanisms. Among these are the acid-sensitive ion channels in the amygdala, which form a likely component of the aversion to CO_2_. When exposure is continued, seizures occur in some animals, soon after the loss of motion in some mice, i.e., at a timepoint where loss of consciousness is uncertain, as demonstrated by EEG and EMG [96]. Nociceptor density in the nasal mucosa is similar to humans who experience discomfort at CO_2_ levels around 30%–35% [52], and it is therefore presumed to be similarly painful.

Inert gases, notably Ar and N_2_, as well as inhalant anaesthetics have been studied as alternatives to CO_2_ [37,39,41], but controversy exists about the relative degree of distress caused by each agent. Currently, LAPS is under investigation as an alternative CAS method. Other inert gases (e.g., helium, xenon) as well as carbon monoxide and nitrous oxide, which have been investigated as potential alternatives to CO_2_ [96], will not be covered by this research strategy due to concerns with operator safety (nitrous oxide, carbon monoxide) or because gases are expensive/not easily accessible, and substantial engineering would be required to create cost-effective systems (helium, xenon). Therefore, it is unlikely that systems would be implemented even if a potential animal welfare advantage over CO_2_ could be demonstrated.

Additional to CAS methods, barbiturate overdose and physical methods are currently the most common methods [7]. These are suitable for single animal use, depending on the experimental regime, but their refinement will not be discussed, as this strategy focuses on methods that are suitable for larger numbers of animals.

An important consideration for the development and evaluation of stunning and killing methods is that in both mice and rats, strain and sex differences are reported for sensitivity to general anaesthetics [123], the avoidance of perceived risks [124], and CO_2_ stunning [40,109]. Any potential alternative method consequently needs to be tested in different strains and both sexes. Additionally, stunning and killing methods must be validated for specific age groups, as physiological responses in new-born animals may differ considerably from adult animals (e.g., higher resistance to hypoxia and hypercapnia [125,126,127,128]).

### 4.2. Rodents-Strategy

#### 4.2.1. Identifying Less Aversive Controlled Atmosphere Stunning Methods

Statement of the Problem

Conceptually, CAS techniques are attractive because they allow stunning multiple animals at a time without restraint and with minimal direct interaction, which is aversive to mice [129,130]. They also kill animals without gross anatomical damage (unlike some physical methods), which may be important for some scientific purposes; however, physiological disturbances may reduce tissue viability and yields [131]. The majority of CAS alternatives have focused on inert gases which, unlike CO_2_, do not have narcotic properties at normobaric conditions (the notable exception being xenon [132]). Hypoxia per se may be aversive to rodents [37,115], and the duration and extent of hypoxia before loss of consciousness in mice exposed to inert gases is significantly increased compared to CO_2_ [41]; therefore, the overall difference in distress caused by the two methods is unknown.

Research Priorities

1 Assess the welfare impact of hypoxia-inducing stunning methods.

Argon: When concentrations below those required for stunning are included, studies in mice and rats suggest that Ar is less aversive than CO_2_ based on voluntarily tolerated exposure times [38,120]. However, rats and mice consistently avoid Ar exposure in approach–avoidance testing when residual oxygen concentration is below 7% (rats) or 9% (mice), i.e., higher than that required for stunning [37,39,115]. On one hand, gasping and seizure-like behaviour was observed in rats prior to recumbency [94], and hyperreflexia was observed when the animals became recumbent [31]. On the other hand, seizures occurred in mice significantly after recumbency near the point of isoelectric EEG. Furthermore, EEG patterns prior to loss of consciousness were indicative of cortical depression (rather than arousal as with CO_2_ [96]). Whether this represents a species difference or variability is not clear. It further remains unclear whether the observed aversion is due to Ar per se, or if it is a response to all hypoxic environments. The level of distress experienced during stunning with Ar relative to CO_2_ remains to be fully characterised in both species, and comparison with N_2_ is also likely to be informative.

Nitrogen: Studies in mice suggest that N_2_ is less aversive than CO_2_ based on locomotor activity, jumping and freezing behaviour, and EEG patterns indicative of cortical depression rather than arousal [41,96]. While seizures were only observed in mice at the onset of isoelectric EEG, hyperreflexia was reported in rats at the timepoint of loss of movement [31]. It is currently unknown whether this hyperreflexia represents neuromuscular dysfunction due to hypoxia or if it is a sign of aversion leading to distress. There is a general lack of data on aversion to N_2_, and suitability is generally inferred from studies investigating Ar, assuming that the degree of hypoxia is predictive for the degree of distress experienced. However, Ar has a higher density than N_2_, and rats survive the same levels of hypoxia longer when air is replaced by N_2_ rather than Ar, which is supposedly due to the lower work of breathing during hypoxia-induced hyperventilation [133]. In rats exposed to N_2_, heart rates were lower than when exposed to Ar (but not CO_2_) suggesting different physiological effects, although it is not clear if this indicates a different degree of stress [31].

Low Atmospheric Pressure Stunning: LAPS is currently being investigated in pilot studies in rodents, and has been approved for the stunning of poultry [9]. Humans are frequently unaware of the onset of hypobaric hypoxia occurring over 2–3 min and require specific training to recognise it [134]. Rodents may respond differently to hypobaric hypoxia than humans, especially given their increased sensitivity to hypoxia [135]. Aversion caused by LAPS should be determined for a thorough assessment of the welfare impact. Optimal rates and timing of decompression also have to be determined. The development of reliable, affordable, easy-to-use systems for routine use would be a prerequisite for the broader implementation of LAPS.

2 Systematically assess the welfare impact of inhalant anaesthetics for stunning in previously exposed animals. Rodents frequently undergo general anaesthesia for experimental procedures. Increased aversion to secondary exposure to isoflurane occurs in mice [119,123] and rats [136,137], and increased aversion to secondary exposure was also demonstrated for sevoflurane in rats [136]. Importantly, rats were able to generalise a learned aversion to one inhalant anaesthetic compared to a novel inhalant anaesthetic [138]. Therefore, aversion to stunning with isoflurane or sevoflurane depends on prior experience. Stunning with isoflurane or sevoflurane may involve a period of behavioural excitation not observed with CO_2_; however, post-mortem catecholamine levels are lower than with CO_2_ [61,123], indicating reduced stress. Thus, the advantages of volatile anaesthetic use for stunning is debated.

Differences in aversion caused by sevoflurane and isoflurane in rats and mice is controversial, with some studies finding less aversion with sevoflurane [139,140], but others finding similar levels [61,136]. The effect of the interval between exposures on the development of increased levels of aversion and the duration of the increased sensitivity is unknown. Similarly, whether aversion continues to increase if the number of exposures exceeds two is unknown. This should be investigated in both species for both isoflurane and sevoflurane in order to quantify the expected distress caused by inhalant anaesthetics. Furthermore, modulating effects of the flow rates, carrier gases (O_2_ versus air), and N_2_O admixture on time to loss of consciousness and displayed aversive behaviours remain to be fully characterised. Strategies to reduce the development of aversion should be investigated, not only for the potential use of inhalant anaesthetics for euthanasia, but also to reduce stress in animals undergoing repeated inhalant anaesthesia for experimentation. In all applications, the correct scavenging of inhalant anaesthetics is important, because halogenated ethers are pollutants, and exposure to operators carries health risks.

3 Optimise flow rates and gas mixtures. While some work has been done to optimise CO_2_ flow rates (gradual fill CO_2_ at 30% is recommended in rats [141], whereas a faster fill rate may be preferable in mice based on behavioural measurements [32,41,63]), little or no data is currently available for optimising flow rates with inert gases [41] or inhalant agents, nor for optimising decompression rates for LAPS.

#### 4.2.2. Identify Suitable Stunning Methods for Neonatal Rodents

Statement of the Problem

Animals under 2 weeks of age are more resistant to hypercapnia and hypoxia than older animals, and therefore cannot be reliably stunned and killed by CAS methods. In Switzerland, the decapitation of conscious animals and immersion in liquid nitrogen of mice up to 2 days of age are both currently allowed. Both techniques kill animals without stunning and may be painful. The consciousness and ability of neonatal rodents to experience pain are debated, but European legislation assumes that mammals can experience suffering from the last trimester of gestation [7]. Therefore, methods to reliably stun neonatal animals prior to killing should be identified.

Research Priorities

1 Investigate the suitability of inhalant anaesthetics for stunning neonatal rodents.

Anaesthesia has been successfully induced with halothane, isoflurane, and sevoflurane in neonatal rodents, although higher concentrations are required than in adult animals [142]. The level of aversion to volatile agents in neonates should be determined.

#### 4.2.3. Develop Reliable, Restraint-Free Physical Methods

Statement of the Problem

Conventional physical methods, i.e., cervical dislocation and decapitation, require restraint of the animal, have high failure rates (around 20% for cervical dislocation [143]), and face ongoing controversy about consciousness after force application, as the brain is not directly targeted [7,12,144]. In many European countries, cervical dislocation and decapitation are prohibited in unanaesthetised animals with the exception of decapitation in rats and mice younger than 2 weeks [22]. For the stunning of larger animals, e.g., cattle, captive bolts are a widely accepted stunning method (which induces unconsciousness by concussion [145]), and an automated captive bolt trap to kill wild rats and mice is commercially available (e.g., the Goodnature trap [146]). However, to date, no feasible application for killing several animals within a short time—all while minimising restraint—is available.

Research Priorities

Adapt captive bolt for use in the laboratory. Automated captive bolt units have been used to attract wild rats and mice using food. When animals rear to eat food in the device, a captive bolt is automatically released, and the animal is killed. While the exact timing doesn’t matter when killing wild rodents, the ability to schedule killing is a prerequisite for any method used in the laboratory. There should be a strong incentive for the rodent to enter the automated device, preferably not requiring prior conditioning. Another factor to be considered is that other animals should not observe what happens upon interaction with the device as rats are able to learn from observations [147], and are capable of causal reasoning [148]. Additionally, alarm calls [149] or pheromones [150,151] emitted upon stunning may cause stress in subsequent animals. Several practical questions remain regarding the implementation of a captive bolt in the laboratory setting to minimise welfare impact, including failure rates. In contrast to decapitation, which leaves the brain (physically) intact, captive bolt techniques have a direct impact on the brain, and are not suitable for experiments investigating the brain post-mortem.

## 5. Poultry

### 5.1. Poultry-Introduction

CO_2_ stunning and electrical water-bath stunning are the two most commonly used methods for rendering broilers unconscious prior to slaughter. Additionally, mobile CO_2_ containers are used to kill low economic value laying hens on-farm at the end of the laying period, or in cases of disease outbreaks [152]. CAS methods were originally introduced with the intention of improving animal welfare by avoiding the shortcomings of electrical water bath stunning, notably the shackling of live birds, the risk of painful electrical shocks, and ineffective stunning in a proportion of animals [28], which may also affect meat quality [153]. However, with CO_2_ stunning headshaking [17,44,56,57,58,59], wing flapping [1,58], mandibulation [1,154], and abnormal respiratory patterns [17,44,56,57,58,59] are commonly observed prior to loss of consciousness. Although the reliability of those behaviours as indicators of distress is debated for individual parameters, their summation is generally interpreted as a relevant level of distress being present.

Inert gas stunning with Ar or N_2_ is permitted, but whilst there is equipment in the market available, it is currently not commercially used in Europe. Low atmospheric pressure stunning (LAPS) was recently approved for the stunning of broilers up to 4 kg in the European Union, as it was judged as “at least equivalent, in terms of animal welfare outcomes, to currently available stunning methods” and appears to result in acceptable meat quality [9,155]. In parallel, high-throughput head-only electrical stunning is being developed as an alternative to conventional multi-bird water bath stunning to prevent pre-stun shocks and insufficient current delivery as well as to improve meat quality [156,157]. For the on-farm killing of larger groups, rapidly expanding gas-filled foam has been verified as a quick and effective method to kill birds directly in the barn [158]. Practical issues with its application (i.e., the over-production of water and logistics of delivering the foam) need further refining to make it commercially viable.

Poultry kept for the production of meat or eggs vary in body size, age, physiology, and behaviour when they are stunned (e.g., laying hens versus broilers versus turkeys). Therefore, the level of aversion caused by a specific stimulus and the behavioural response to a comparable level of experienced aversion may vary [157]. Additionally, differences in age as well as body size and composition are relevant for the effectiveness of some stunning techniques; for example, chicks are more resistant to CO_2_ than older animals, and require lower pressure to be killed by LAPS [159], and maximal times to motionlessness were higher in LAPS studies investigating Ross broilers than in studies investigating Cobb broilers, whether it was due to genotype, bodyweight, or the age of the different study populations [9]. Therefore, the suitability of a stunning method needs to be assessed for each production type, age group, strain, and breed separately, keeping in mind that genetics are constantly evolving within the breeding industry.

### 5.2. Poultry-Strategy

#### 5.2.1. Identify Less Aversive Controlled Atmosphere Stunning Methods

Statement of the Problem

CAS techniques are attractive because they can be performed with birds remaining in the transport cage, and avoid the shackling of birds prior to stunning, which is stressful and may be painful [160]. However, CO_2_ is aversive to poultry [44,45,46,161], activates nociceptors in the nasal mucosa of chickens [55], and leads to behaviours that are generally interpreted as signs of distress or air hunger [1,56,59,162]. Different CAS methods should be identified to reduce distress, and tools should be established to allow comparisons between methods/techniques.

Research Priorities

1 Systematically assess the welfare impact of hypoxia-inducing stunning methods at optimal flow or decompression rates. CAS methods induce unconsciousness by inducing hypoxia. Studies generally indicate that Ar or N_2_ are less aversive than CO_2_ [44] and delay the time to onset of aversive behaviours [35,56], as well as that birds stunned with Ar show less abnormal respiratory patterns than with CO_2_ [1,17,56,59]. The meat quality of broilers stunned with 90% Ar was similar to that of broilers stunned with biphasic CO_2_ exposure [1].

However, comparisons of CO_2_ and LAPS as well as N_2_ and LAPS have only been performed in day-old male chicks so far [159]. Cortisone and serotonin levels did not significantly differ between the three stunning methods, but headshaking and gasping prior to loss of consciousness were significantly less common with N_2_ and LAPS than with CO_2_, with no significant difference between N_2_ and LAPS. With both hypoxia methods, time to loss of posture was significantly longer than with gradual fill CO_2_, and time to loss of posture with gradual fill of N_2_ was significantly longer than with LAPS. As chicks are more resistant to CO_2_ than older animals and require lower pressures to be killed by LAPS [159], it remains to be determined whether differences exist between hypoxic stunning methods and if LAPS results in reduced distress compared to CO_2_ in older birds.

Optimal rates of decompression have been determined for LAPS in broilers up to 4 kg, but the suitability of the method for other production types and species remains to be investigated, as well as optimal decompression rates.

Due to their respective densities, Ar and N_2_ are suitable for immersion and gradual fill designs, respectively. However, there is no data available regarding whether immediate exposure to target concentrations or gradual fill with the same gas elicit different levels of aversive behaviours and abnormal respiratory patterns prior to loss of posture.

2 Develop alternatives for large-scale on-farm killing. As an alternative to currently used mobile CO_2_ containers, the development of mobile N_2_ or Ar containers [163] or LAPS units should be investigated. However, mobile units require that animals are caught and carried out of the barn prior to stunning, which is a major source of stress.

Whole-house gas-killing methods would avoid the stress of capture and restraint. Anoxic gas mixtures could be delivered by high-expansion foam filled with N_2_ (or other gases), which—due to large bubble size—do not occlude the airways in an experimental setting (in contrast to less expansive foams), but locally creates an atmosphere containing only 1–2% O_2_ [164]. Further research is needed to test their feasibility in commercial settings.

#### 5.2.2. Improve Electrical Stunning Techniques and Investigate Alternatives Directly Targeting the Brain

Statement of the Problem

Electrical stunning traditionally induces unconsciousness by causing generalised seizures. Conventional multi-bird electrical water bath stunning has several shortcomings, such as the need for shackling, the risk of painful shocks, and the risk of ineffective stunning resulting in consciousness during neck cutting [28,165]. However, electrical stunning does have the advantage of providing almost immediate unconsciousness (within a second) when sufficiently high currents are delivered [166]. Attempts have been made to increase the efficacy of the stunning with a head-cloaca stunning system, which ensures that the full current is delivered to each bird once contact with the water bath is made [167], and individual head-only stunning [156]. For the head-only stunning, the shackling of live birds is replaced with upside-down restraint in a plastic cone, which allows movement of the head and results in birds voluntarily exposing their head in the required position as they try to place it horizontally. Birds were effectively stunned, and meat quality was superior compared to conventional water-bath stunning [156]. Systems are now commercially available (e.g., [168]). However, the duration of unconsciousness induced by head-only stunning may be insufficient [157].

Research Priorities

1 Develop electrical stunning techniques that immediately and reliably stun birds of all sizes, strains, and ages, while minimising handling and restraint. Even with the head-only stunning, birds still need to be handled, restrained, and turned upside-down in conventional cones. This could be improved by the use of an inclined rather than inverted cone, as suggested by Gregory and Gibson [157]. For shackling, it has been shown that the duration of shackling positively correlates with cortisone levels and stress levels, as indicated by the tonic inhibition test [169]. Restraint systems and set-ups for current delivery should be further refined to minimise handling and restraint, including the duration of restraint, while still reliably stunning all types of birds. Furthermore, alternative voltage and current settings should be investigated, especially for head-only stunning, to ensure that the duration of the stun lasts until death. For example, a single-pulse ultra-high current (SPUC) uses a substantially higher current than conventional electrical water-bath or head-only stunning and successfully induced unconsciousness for up to four minutes in cattle without causing seizure activity [145].

2 Compare aversion between optimised electrical and CAS stunning techniques. Electrical stunning methods and CAS differ in the type of aversive stimulus; electrical stunning requires handling and—to date—restraint in an upside-down position, whereas CAS requires an extended period of exposure to unphysiological ambient conditions. The differences in distress resulting from restraint compared to pre-stun CAS periods are unknown, and should be investigated.

## 6. Pigs

### 6.1. Pigs-Introduction

The two most common methods of stunning pigs prior to slaughter are electricity and CO_2_ [170]. The development of CO_2_ stunning was introduced to reduce stress related to social isolation and the use of electrical prods to separate animals, which is traditionally required for electrical stunning. Physiological variables (plasma lactate) have been correlated to behaviours indicative of aversion (vocalisation, falls, rearing, and backing up), and concluded that group pens were less aversive than single file races [171,172]. Such data is complicated by the subsequent stunning paradigm as blood is collected post mortem. Furthermore, neither physiological variables nor the cited behaviours are validated measures of distress in pigs.

There are several reports of aversive behaviours in pigs exposed to various concentrations of CO_2_, as low as 15% [27,42,43,171,173]. The inert gas Ar used at 90% reduced aversive behaviours during stunning; however, it still resulted in some aversion [43]. No studies of high concentration N_2_ stunning have been published in pigs, despite suggestions from data in rats that it may result in reduced stress responses compared to Ar [31].

### 6.2. Pigs-Strategy

#### 6.2.1. Determine Aversiveness of Inert Gas Hypoxia

Statement of the Problem

Hypoxia resulting from displacement by 90% Ar results in less aversive behaviour than either 15% or 30% CO_2_ with N_2_ as a filler gas [43]. This suggests that inert gas hypoxia may offer a welfare improvement over CO_2_ containing mixtures, but is not non-aversive. The stunning of animals by hypoxia can be achieved by three primary methods: gas displacement, immersion into gas, and low atmosphere pressure stunning (LAPS), all of which lower the partial pressure of O_2_ in the chamber until it is insufficient to support consciousness. Times required to ensure death in pigs immersed in N_2_ and Ar are around 7 min compared to 3 min for CO_2_, suggesting a longer period of conscious exposure. The degree of aversion and distress during this period is unknown. There are currently no published studies on aversion to LAPS in pigs. Recurring behavioural findings of inert gas displacement hypoxia across species are “air hunger” and “gasping”, which are characterised by heavy open mouth breathing with exaggerated thoracic movement. This is frequently assumed to indicate distress; however, no direct evidence exists to support this.

Research Priorities

1 Compare LAPS to N_2_, Ar, and CO_2_ to determine if hypoxic aversion is reduced. Inert gas hypoxia has been shown to be less aversive than CO_2_ exposure [43]; however, there is currently no data on aversiveness to LAPS-induced hypoxia. Given the financial investments required to establish CAS methods for pigs, establishing which method is least aversive is important.

2 Improve process engineering to consistently produce and maintain an anoxic (<2% volume O_2_) atmosphere in commercial stunning and euthanasia equipment. Effective inert gas stunning requires sustained ambient O_2_ concentrations under 2%. Increases to 6% may result in spontaneous recovery [42,173]. Since N_2_ is not sufficiently dense to use in dip-lift systems, sealed chambers are required to achieve hypoxic conditions. However, Ar mixtures may be used in dip-lift systems [43]. Furthermore, such systems should be capable of inducing anoxia in an appropriate timeframe, which is yet to be determined. Insufficient filling rates are likely to result in prolonged stress before loss of consciousness.

#### 6.2.2. Comparison of Physical Stunning Methods

Statement of the Problem

Electrical stunning is considered to render pigs unconscious immediately and is a commonly used technique prior to slaughter [174]. The main problems with this stunning technique are that seizures may occur, which can pose a danger to operators; unconsciousness may be short lived, risking the chance of regaining consciousness before bleeding has been completed; and pigs are commonly separated from their group to load into a stunning pen. This latter point poses several welfare issues, namely that electrical prods may be used to load the animals, social isolation may be distressful [175], and the nature of the stunning pen may be aversive to the pigs. One form of restraining device for electrical stunning has been suggested to be equally aversive as exposure to 90% CO_2_, and the electrical prodder required for loading into the restrainer was more aversive [112]. The issue of isolation can be overcome by using group stunning pens. Then, the electrical stun is applied by an operator using hand-held tongues. This can induce more variability in the stun efficacy and lengthen the time from stunning to exsanguination, therefore increasing the risk of regaining consciousness [176]. One alternative to conventional electrical stunning is the application of single-pulse ultra-high current (SPUC) stunning, which has been demonstrated to increase times of unconsciousness compared to conventional stunning in cows [145]. Furthermore, no epileptiform activity is reported with this method. Group stun pens may increase anxiety spreading between individuals; therefore, the advantage over single-chute systems is unclear. A further refinement to group stun pens could be to use alternative stunning methods that allow pigs to be stunned together, in the same way as with gas. Exploring whether microwave radiation would be effective could be an option.

This method rapidly heats the brain to induce insensibility in rats and unconsciousness in cattle [177]. This may be applied using a hand-held device and only requires one point of contact, unlike electrical stunning tongues.

Research Priorities

1 Evaluate distress in group pen and isolation stunning paradigms. Pigs suffer from isolation stress; however, a single animal exhibiting aversion-related behaviours may result in such behaviours being exhibited by others in the group. The proposed benefits of electrical stunning in groups remains inconclusive since they are kept in groups, they are individually restrained at the point of stunning, and stunning is staggered within the group in sight of other pigs.

2 Develop techniques for more accurate electrical stunning without the need for restraint. Electrical stunning may be performed in group pens, although this relies on partial individual restraint at the point of stunning and the manual placement of stunning tongues by an operator. This may result in less accurate placement and therefore inadequate stunning, and also exposes animals to unfamiliar humans, which is considered a stressor. One refinement to this might be the use of SPUC to induce a more rapid and longer duration loss of consciousness [145], which should be tested in pigs. The feasibility of using SPUC in a group pen situation would also be of value.

## 7. Conclusions

Here, we suggest a series of research priorities that are intended to identify humane alternatives for the stunning of mice, rats, poultry, and pigs. The aims detailed in this document are not an exhaustive list of avenues to explore, but rather the methods that offer the most promising opportunity for practical implementation. Our proposal is not based on any political or legislative landscape, and as such, opens an avenue for international co-operation toward improving animal welfare by humanely ending the life of animals.
